# Supplementary material for: Effects of Myofascial Release Techniques on Joint Range of Motion of Athletes: A Systematic Review and Meta-Analysis of Randomized Controlled Trials
Source: Sports (Basel). 2024 May 14;12(5):132. doi: 10.3390/sports12050132 (PMC11125680; doi:10.3390/sports12050132)
Supplement: Supplementary file 1 [file sports-12-00132-s001.zip › sports-2871194-supplementary.pdf]

Table S1. Coding strategy and lines of code used in databases.

Databases	Keywords	Number of Articles Accessed	Search Restriction Criteria
**Pubmed**	(Myofascial release [MeSH Terms] OR Myofascial Release Therapies OR Therapy, Myofascial Release OR- Myofascial Release Treatments OR Treatment, Myofascial Release OR Myofascial Treatments OR Treatment, Myofascial)<br>AND<br>(range of motion[title] OR Joint ROM OR flexibility OR passive range of motion OR active range of motion)<br>AND<br>(Athletes OR Professional Athletes [MeSH Terms] OR Elite Athletes OR College Athletes)	34	Title/abstract
**Science Direct**	"Myofascial release" OR "Myofascial Release Therapies", Myofascial, "Myofascial Release" OR "Myofascial Treatments") AND ("range of motion" OR "Joint ROM" OR flexibility) AND (Athletes OR "Professional Athletes"	237	Research articles
**Web of Science**	"Myofascial release" OR "Myofascial Release Therapies", Myofascial, "Myofascial Release" OR "Myofascial Treatments") AND ("range of motion" OR "Joint ROM" OR flexibility) AND (Athletes OR "Professional Athletes"	45	Topic
**Scopus**	"Myofascial release" OR "Myofascial Release Therapies", Myofascial, "Myofascial Release" OR "Myofascial Treatments") AND ("range of motion" OR "Joint ROM" OR flexibility) AND (Athletes OR "Professional Athletes"	47	Title/abstract/keyword
**Cochrane**	""Myofascial release" OR "Myofascial Release Therapies", Myofascial, "Myofascial Release" OR "Myofascial Treatments") AND ("range of motion" OR "Joint ROM" OR flexibility) AND (Athletes OR "Professional Athletes"	48	Title/abstract/keyword
**TOTAL NUMBER OF ARTİCLE ACCESSED**		411	

Table S2. The potential moderators of this study and coding categories.

Age	Gender	Duration of intervention	Type of intervention	Joint type
<25 years of age<br>≥25 of age	Male<br>Female	Acute<br>Long-term	1.Myofascial release methods (foam rolling // fascial manipulation // jogging + foam rolling // DN with water pressure massage—placebo laser with water pressure massage // self-myofascial release [foam rolling])<br><br>2. Instrumental methods (IASTM // dry needling)<br><br>3. Stretching technique methods (Contract-relax PNF stretching technique)<br><br>4. Other methods (local MTrPs therapy (myofascial trigger points) + PNF stretching technique)	Hip<br>Ankle<br>Knee<br>Cervical<br>Shoulder

Table S3. Quality assessment (PEDro scale) of clinical trials included in the systematic review.

Authors	1*	2	3	4	5	6	7	8	9	10	11	Total PEDro score
Romero-Franco (2019)	1	1	1	0	1	1	0	1	1	1	1	8
Brandolini (2019)	1	1	1	0	0	0	1	1	1	1	1	7
Castellote-Caballero (2013)	1	1	0	1	1	0	1	1	1	1	1	8
Guillot (2019)	1	1	1	0	0	1	0	1	1	1	1	7
Weber (2022)	1	1	1	1	0	0	1	1	1	1	1	8
Junker & Stöggl (2019)	1	1	1	0	0	0	0	1	1	1	1	6
Abo-EL-Roos (2020)	1	1	0	1	1	0	0	0	1	1	1	6
Ceballos-Laita (2021)	1	1	1	0	0	1	1	1	1	1	1	8
Haser (2017)	1	1	0	1	1	1	0	1	0	1	1	7
Shalamzari (2022)	1	1	0	0	1	1	0	1	0	1	1	6

Note1. 1:Yes,0:No.
Note 2. *: Criteria 1 does not contribute for the total score on the PEDro scale.
Note 3. Criteria 1: Eligibility criteria; Criteria 2: randomized allocation; Criteria 3: concealed allocation; Criteria 4: similar at baseline; Criteria 5: blinded subjects; Criteria 6: blinded therapists; Criteria 7: blinded assessors; Criteria 8: measures of at least one key outcome obtained for 85% of subjects; Criteria 9: intention-to-treat analysis; Criteria 10: between-group comparisons for at least one key outcome; Criteria 11: point and variability measures for at least one key outcome.

Table S4. Sensitivity analysis results for dependent effect size estimation with different RHO (ρ) values.

	$\rho = 0$	$\rho = 0.2$	$\rho = 0.4$	$\rho = 0.6$	$\rho = 0.8$	$\rho = 1$
Coefficient	0.53	0.53	0.53	0.53	0.53	0.53
SE	0.15	0.15	0.15	0.15	0.15	0.15
τ^2	0.58	0.58	0.58	0.59	0.59	0.59

Note. SE: Standard error.

Table S5. Evaluation of certainty of evidence using the GRADE scale.

Outputs	Risk of bias	Inconsistency	Indirectness	Imprecision	Publication bias	Certainty of evidence
Overall effect (ROM performance)	Not significant	Not significant	Significant	Not significant	Not significant	⊕⊕⊕◯ Moderate [a]
Moderator analysis (age)	Not significant	Not significant	Significant	Not significant	Not significant	⊕⊕⊕◯ Moderate [a]
Moderator analysis (gender)	Not significant	Not significant	Significant	Not significant	Not significant	⊕⊕⊕◯ Moderate [a]
Moderator analysis (intervention duration)	Not significant	Not significant	Significant	Not significant	Not significant	⊕⊕⊕◯ Moderate [a]
Moderator analysis (intervention type)	Not significant	Not significant	Significant	Not significant	Not significant	⊕⊕⊕◯ Moderate [a]
Moderator analysis (joint type)	Not significant	Not significant	Significant	Not significant	Not significant	⊕⊕⊕◯ Moderate [a]

[a] Downgraded by one level because the study result contains a high degree pf heterogeneity (>75%).

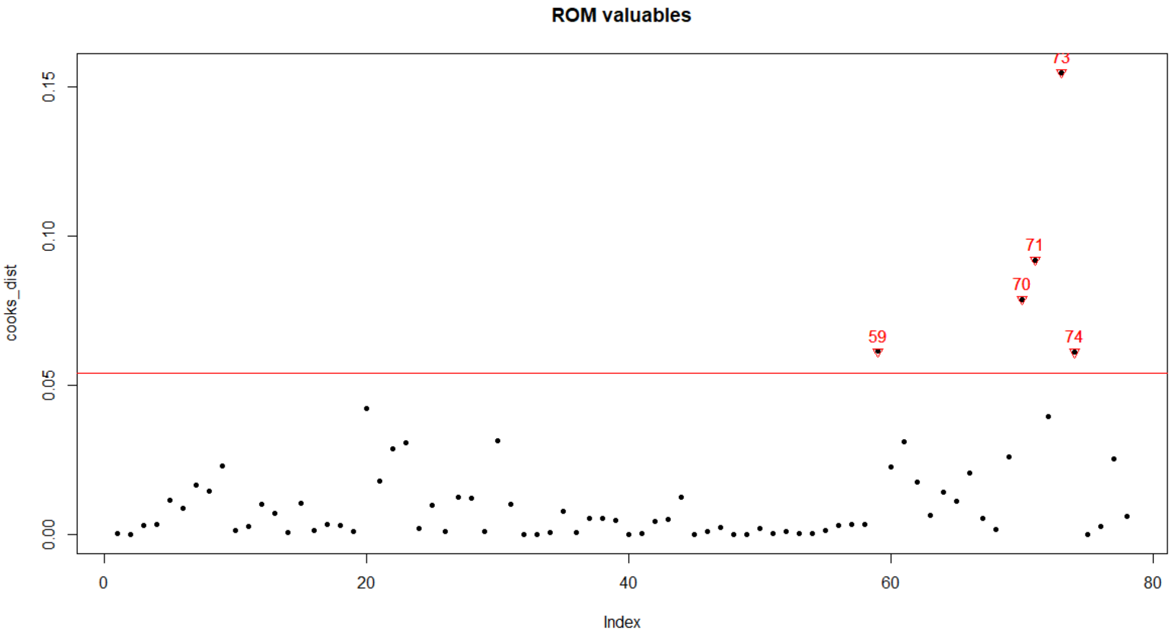

Figure S1. Identifying outliers using Cook's distance analysis.
